# Intensity of ^18^F-FDG PET Uptake in Culture-Negative and Culture-Positive Cases of Chronic Osteomyelitis

**DOI:** 10.1155/2017/9754293

**Published:** 2017-10-11

**Authors:** Petteri Lankinen, Marko Seppänen, Kimmo Mattila, Markku Kallajoki, Juhani Knuuti, Hannu T. Aro

**Affiliations:** ^1^Orthopaedic Research Unit, Department of Orthopaedic Surgery and Traumatology, Turku University Hospital, University of Turku, Turku, Finland; ^2^Turku PET Centre, Turku University Hospital, Turku, Finland; ^3^Medical Imaging Centre of Southwest Finland, Turku University Hospital, University of Turku, Turku, Finland; ^4^Department of Pathology, Turku University Hospital, Turku, Finland

## Abstract

Microbiologic cultures are not infrequently negative in patients with a histopathologic diagnosis of chronic osteomyelitis. Culture-negative cases may represent low-grade infections with a lower metabolic activity than culture-positive cases. ^18^F-FDG PET could potentially detect such a difference. We determined whether the level of ^18^F-FDG PET uptake differs in patients with culture-negative and culture-positive osteomyelitis. We reviewed the clinical charts of 40 consecutive patients, who had diagnostic ^18^F-FDG PET for a suspected bone infection. Twenty-six patients were eligible with a confirmed diagnosis based on microbiologic cultures and/or histopathologic examination. Sixteen of 26 patients had chronic osteomyelitis. Eight of them had positive cultures, seven had negative cultures, and one patient had no cultures of the biopsy specimen. The patients with histologically and/or microbiologically proven osteomyelitis were correctly interpreted as true positive in the routine clinical reading of ^18^F-FDG PET images. There was no relationship between the level of ^18^F-FDG PET uptake and the presence of positive or negative bacterial cultures. The result favors the concept that that culture-negative cases of osteomyelitis are false-negative infections due to nonculturable microbes. ^18^F-FDG PET may help to confirm the presence of metabolically active infection in these patients and guide their appropriate treatment.

## 1. Introduction

Osteomyelitis, especially its less common hematogenous forms, is a remarkably difficult diagnostic problem. Based on recent meta-analyses [1–3], fluorodeoxyglucose positron emission tomography (^18^F-FDG PET) is the most sensitive radiographic technique for detecting chronic osteomyelitis and it has a greater specificity than leukocyte scintigraphy, bone scintigraphy, or magnetic resonance imaging. ^18^F-FDG PET is less accurate in the diagnosis of periprosthetic joint infections [4]. One of the contributing factors may be the virulence of the causative bone pathogen and the severity of the subsequent infection [5], which appear to contribute to the intensity of local ^18^F-FDG uptake in infected tissues. In a standardized animal model, localized subacute/chronic osteomyelitis caused by* S. epidermidis* has been characterized by a low ^18^F-FDG uptake [6], while acute suppurative osteomyelitis caused by* S. aureus* results in a high uptake [7, 8].

The definitive diagnosis of osteomyelitis is made by culturing the pathogen from the site of infection. Unfortunately, biopsy cultures are not infrequently negative emphasizing the importance of both histologic and microbiologic samples of tissue samples [9]. In children, the rate of culture-negative osteomyelitis has been reported to be up to 47% [10]. The rate of negative cultures in histologically proven cases of osteomyelitis obtained from imaging-guided bone biopsies (excluding spine biopsies) was even higher (66%) [11]. In recent studies of vertebral osteomyelitis, the negative culture rate of image-guided biopsy was also high (68–70%) [12, 13]. The factors that predict positive or negative culture results are largely unknown [11, 14]. One reason is inappropriate culture conditions. Technical errors of biopsies and starting of antibiotic treatment before biopsy may also affect culture results. There are cases in which even repeated open biopsies fail to recover the underlying pathogen. Sequestra of chronic osteomyelitis are known to be covered by metabolically quiescent bacteria within adherent biofilms [15] and it has been suggested that false-negative infections are due to viable but nonculturable biofilm organisms [14].

Based on this knowledge, certain cases of culture-negative, histologically low-grade osteomyelitis may represent clinical conditions with an inherent difference in metabolic activity compared with culture-positive cases. We assumed that ^18^F-FDG PET could potentially detect such a difference, because intracellular accumulation of the tracer reflects metabolic rate of cells at sites of infection and inflammation [16]. Thus, the purpose of this study was to determine whether the level of ^18^F-FDG uptake differs in culture-negative and culture-positive cases of histologically and/or microbiologically proven osteomyelitis.

## 2. Methods

### 2.1. Patients

A part of this study has been published in a Ph.D. work [17]. The patient population represents 40 consecutive orthopaedic patients who had ^18^F-FDG PET in a five-year period (ending December 2004) with minimum 4-year follow-up data as an adjunct imaging modality for evaluation of a clinically suspected bone infection. The suspicion of bone infection was based on clinical symptoms, laboratory findings, and results of other imaging modalities. The study cases were retrieved from the hospital database based on the reference number of the PET imaging. The clinical charts of the patients were retrospectively reviewed. There was no contact with patients, and according to the national law the study did not require approval of the ethical aboard. The investigation was approved by the hospital administration and was conducted in accordance with the principles of Declaration of Helsinki.

Fourteen of the original 40 patients were excluded from the current analysis. Seven cases were excluded because no histopathologic or microbiologic verification of the diagnosis was made. Five cases were excluded because ^18^F-FDG PET was applied only to evaluate of antimicrobial treatment response. Two additional cases were excluded because the primary indication for PET imaging was not suspected infection.

Twenty-six (65%) of the 40 patients had definite histopathologic and/or microbiologic diagnosis based on the examination of samples (Table 1). Biopsy samples were obtained during neurosurgical decompression of the spinal canal, during an open biopsy performed by an orthopaedic surgeon, or by CT/MRI guided needle biopsy performed by a musculoskeletal radiologist. The definite diagnosis was osteomyelitis in 16 patients (62%), soft tissue infection in four patients (15%), and spinal implant infections in two patients (8%). The histologic diagnoses for the four remaining cases were plasmacytoma, osteoblastoma, transient bone marrow oedema, and degenerative sternoclavicular osteoarthritis.

A microbiologic culture was considered positive, if any relevant organism grew based on the judgment of a microbiologist. Of the 16 cases with proven osteomyelitis, eight (50%) had positive cultures and seven (44%) had negative cultures (Table 2). One patient with low-grade sacral osteomyelitis (case #4) had no cultures done of the biopsy specimen. The culture-negative cases were predominantly rare forms of hematogenous osteomyelitis, including two indolent Brodie's abscesses, two cases of recurrent chronic osteomyelitis of the femur, one case of chronic osteomyelitis of the medial clavicle (possible synovitis, acne, pustulosis, hyperostosis, and osteitis (SAPHO) syndrome), and two cases of vertebral osteomyelitis (Table 2).

None of the patients with negative cultures had antibiotic therapy before sampling (Table 2). Three patients with negative cultures had repeated biopsies because of failures in recovering the pathogen (Table 2). Three other patients with culture-negative osteomyelitis had multiple tissue samples taken at surgery (laminectomy) or during open biopsies. Only one case of culture-negative vertebral osteomyelitis was based on a single procedure of CT-guided biopsy. Routine microbiologic analysis of bone specimens included extended culture times and specific cultures for the diagnosis of tuberculosis, if indicated. Molecular diagnostic technology of polymerase chain reactions (PCRs) was applied in four patients with repeated biopsies (in three culture-negative patients and one culture-positive patient). None of universal PCRs were positive.

There were three patients with a past history of a skeletal infection with fever in both groups (culture-negative cases #2, #5, and #30 and culture-positive cases #6, #16, and #40). Two of these cases had history of osteomyelitis treatment as a child. One additional culture-positive case (case #8) had been hospitalized for an unexplained skeletal pain as a 15-year-old. The current diagnostic studies were most commonly started due to local pain or night aching. None of the patients had fever or draining sinus as a sign of acute exacerbation of chronic osteomyelitis. Pain had lasted ≤ one month in six patients and for 3–12 months in the remaining 10 patients. Aside from pain, one patient had recognized a local resistance (case #3). One patient was symptomless (case #8). One patient (case #20) suffered from radiating back pain for a month and developed acute paraparesis before spinal decompression.

Patient groups of culture-negative and culture-positive osteomyelitis both had normal or slightly elevated blood levels of C-reactive protein (median 6 mg/L, range 1–73 mg/L, respective to median 6 mg/L, range 1–49 mg/L). All patients with culture-negative osteomyelitis, except one, had slightly elevated erythrocyte sedimentation rate (median 18 mm/hour, range 7–27 mm/hour). There was a trend for higher erythrocyte sedimentation rate (median 28 mm/hour, range 2–82 mm/hour) in culture-positive cases.

### 2.2. ^18^F-FDG PET

^18^F-FDG PET imaging was performed as an adjunct part of routine work-up in the differential diagnostics of osteomyelitis. The clinical reviewers of the PET images had access to all patient charts, including the results of conventional imaging modalities. Based on the interpretation of the reviewers, the result of ^18^F-FDG PET was recorded as true positive or false negative (Table 3). The patients were instructed to fast for 6 hours prior to PET. ^18^F-FDG PET imaging was performed with an Advance PET scanner (General Electric Medical Systems, Milwaukee, WI, USA) operated in 2-dimensional mode (high resolution). The scanner had 18 rings of bismuth germanate detectors, and the axial length of the imaging field of view (FOV) was 152 mm. Whole body acquisition was started 60 minutes after the injection of ^18^F-FDG (5 minutes per bed position). The mean dose of intravenous bolus injection of ^18^F-FDG was 297 MBq (SD 71 MBq; range, 160–384 MBq). A standard transmission scan for attenuation correction was obtained after the emission imaging using two rod sources containing germanium-68. All 35 transaxial image slices were reconstructed with an ordered subsets expectation maximization algorithm (OS-EM) and the central 200 mm-diameter transaxial FOV and 128 × 128 matrix leading to pixel size 1.56 × 1.56 mm were used. Random counts and dead time were corrected by the system and scatter correction was incorporated into the reconstruction algorithm. Quantitative analysis of the ^18^F-FDG uptake was performed on standardized circular regions of interest (ROIs, diameter of 15 mm) at the site of visually detected increased tracer accumulation from background using transaxial slices. Tracer accumulation was reported as the standardized uptake value (SUV), which was calculated as the radioactivity of the ROI divided by the relative injected dose expressed per patient's body weight. Both SUV_mean_, representing the average uptake on the selected ROI, and the maximum SUV value (SUV_max_), representing the highest pixel uptake in the ROI, were analyzed. In addition, SUV_ratio_, that is, the ratio between the site of suspected infection and the ROI of the corresponding healthy anatomic site, was calculated for SUV_mean_ and SUV_max_ [7, 18].

### 2.3. MRI, Bone Scintigraphy, and Infection Scans

The decision to perform other imaging modalities (MRI, three-phase bone scintigraphy, infection scan with labeled leukocytes or antigranulocyte antibodies, and occasionally CT) (Table 3) was based on the judgment of clinical indications in each case. The three-phase bone scintigraphy (bone scan) was performed with ^99m^Tc-HDP or DPD (mean dose 670 MBq). Infection scans were performed using either ^99m^Tc-white blood cell scanning (HMPAO, Ceretec, GE Healthcare, Amersham Place, United Kingdom, mean dose 209 MBq) or ^99m^Tc-antigranulocyte scintigraphy (LeukoScan®, Immunomedics GmbH, Darmstadt, Germany, mean dose 1000 MBq) technique. Based on the interpretation of the clinical reviewers, the results were recorded as true positive or false negative (Table 3).

### 2.4. Statistical Analysis

Data are expressed as mean ± standard deviation (SD). Nonparametric Mann–Whitney *U* test was applied in the comparison of SUV_mean_ and SUV_max_ values between patients with culture-positive and culture-negative osteomyelitis. *p* < 0.05 was considered significant. All statistical analyses were performed using IBM SPSS Statistics software (International Business Machines Corp., Armonk, New York, USA).

## 3. Results

**Figure 1:**
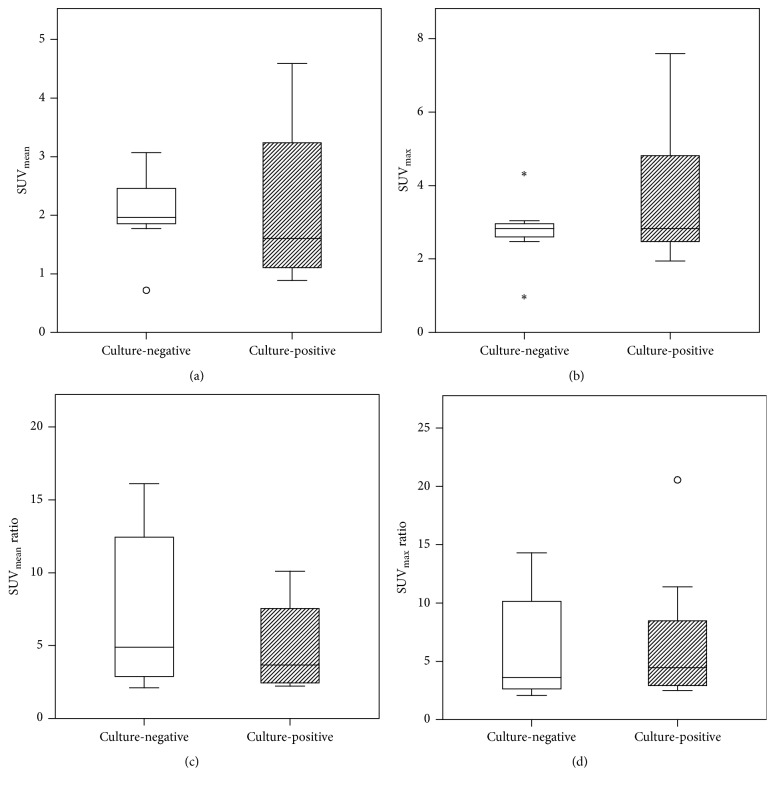
Comparison of SUV_mean_ (a), SUV_max_ (b), SUV_mean_ ratio (c), and SUV_max_ ratio (d) values measured in ^18^F-FDG PET imaging of osteomyelitis patients. The differences between culture-negative (*n* = 7) and culture-positive (*n* = 8) cases were not statistically significant. Box plots are showing median, 1st and 3rd quartiles, minimum and maximum values, and outliers (white circles and asterisks).

SUV_mean_ values of culture-negative (mean ± SD, 2.07 ± 0.74) and culture-positive cases of chronic osteomyelitis (2.18 ± 1.31) did not differ significantly. The SUV_max_ values of culture-negative (2.81 ± 0.96) and culture-positive cases (3.73 ± 1.70) neither showed significant intergroup differences (Figure 1).

**Figure 2:**
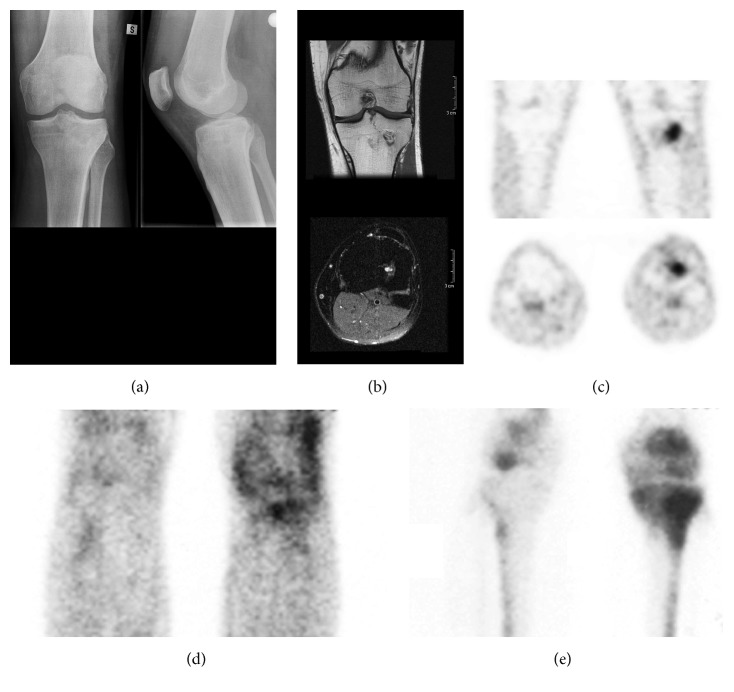
A 25-year-old man with an indolent Brodie's abscess in the proximal tibia (case #8). The patient had been hospitalized for knee pain 10 years earlier, but no specific diagnosis was made. He now suffered a sports related ACL ligament rupture of his left knee. As an incidental finding, anterior-posterior and lateral radiographs (a) showed cystic lesion with surrounding sclerosis in the proximal tibia. Coronal and transaxial MR-images (b) demonstrated a 2 cm sclerotic osseous lesion with contrast medium enhancement and oedema of the surrounding tissues. Coronal and transaxial ^18^F-FDG PET images (c) showed an increased local uptake of the tracer. Compared with the corresponding ROI of the contralateral tibia, SUV_mean_ ratio was 10.1 and SUV_max_ ratio 20.51. The lesion was correctly characterized with infection scintigraphy with labeled antibody fragments (LeukoScan) (d) and three-phase bone scintigraphy (e). Based on percutaneous biopsy samples taken under fluoroscopy, the final histological diagnosis was Brodie's abscess and the microbiologic culture revealed* S. aureus* as the causative pathogen.

There was a clear visual difference in the uptake of ^18^F-FDG at the infection site and at the corresponding ROI of the contralateral healthy bone (Figure 2). Both in culture-negative and culture-positive cases, the calculations of SUV_ratio_ confirmed high mean values for SUV_mean_ (7.65 versus 4.98, resp.) and for SUV_max_ (6.51 versus 6.84, resp.) (Figure 1).

The patients with histologically and/or microbiologically proven osteomyelitis (*n* = 16) were all correctly interpreted as true positive in the routine clinical reading of ^18^F-FDG PET images (Table 3). Four patients (25%) out of 16 (cases #4, #7, #20, and #40) had false-negative MRI or labeled leukocyte scintigraphy (Table 3). In the retrospective view, in these cases^ 18^F-FDG PET brought a significant diagnostic help compared with the results of other imaging modalities.

Among the whole group of patients (*n* = 26) (Table 1), ^18^F-FDG PET gave no false-negative cases and three false positive cases. The three false positive cases were due to periarticular soft tissue infections (cases #12 and #33) and vertebral plasmacytoma (case #26).

## 4. Discussion

The present retrospective analysis was focused on the diagnostic imaging and microbiologic challenges in a special subgroup of patients with predominantly rare forms of hematogenous chronic osteomyelitis, including four cases of Brodie's abscesses. The rate of positive cultures was 47%, which is similar to the reported rates of 30%–42% in previous studies on imaging-guided biopsies with combined histologic and microbiologic evaluation [9, 11–13]. Thus, our patient population resembles the previously published series and was subsequently appropriate for evaluation of ^18^F-FDG PET imaging in the characterization of culture-negative cases. The culture-negative cases may be incorrectly described as negative because the infecting microbe(s) may be nonculturable [14]. If true, we assumed that culture-negative cases might have a lower metabolic activity than culture-positive cases measured by ^18^F-FDG PET imaging. Against our hypothesis, there was no relationship between the level of ^18^F-FDG PET uptake and the presence of positive or negative bacterial cultures among these patients with histologically and/or microbiologically proven chronic osteomyelitis.

Reflecting the rarity of the cases, it is evident that a multicenter prospective study is needed to get definitive answers to the open questions. Previously Wu and coworkers [11] have already paid attention to the small number of requests for imaging-guided core bone biopsies for suspected osteomyelitis. They found that two large US centers had only 3–7 such cases per year. The number of cases enrolled in our study closely resembles the experience of Wu and coworkers. Twenty-six patients, who had ^18^F-FDG PET imaging for suspected osteomyelitis and underwent the necessary microbiologic and/or histologic examinations of biopsies during a five-year period, represented about 5 referred cases per year in our university hospital district of about 800.000 inhabitants. Most of these cases were primarily scrutinized by the sarcoma treatment group for exclusion of a bone tumor. Overall, it is important to emphasize two facts. First, the majority of osteomyelitis patients (like posttraumatic cases) have an undisputed clinical history with clear-cut laboratory/radiographic data suggesting osteomyelitis and do not require advanced noninvasive differential diagnostics like ^18^F-FDG PET imaging and a histologic proof of the diagnosis. Secondly, the microbiologic isolation of the causative bone pathogen(s) yields positive results in most cases (78%) of posttraumatic osteomyelitis [19] and, for example, virtually in all cases with recurrent infection of open tibial fractures [20]. As shown in the previous studies [9, 11–13] and in the current study, the situation is different in subgroups of patients who are referred to ^18^F-FDG PET imaging and/or imaging-guided biopsy for suspected chronic osteomyelitis.

Based on previous studies, a negative ^18^F-FDG PET scan can virtually rule out chronic osteomyelitis [18]. The high accuracy of ^18^F-FDG PET for excluding chronic osteomyelitis may be related to the high uptake of ^18^F-FDG by activated macrophages, which are among the predominant cells in chronic infections [16]. The false positive case of vertebral plasmacytoma demonstrates the inability of ^18^F-FDG PET imaging in the differentiation of chronic osteomyelitis and a malignant bone tumor. Both of these conditions result in the high accumulation of ^18^F-FDG and may even share the macrophage-related mechanism of the tracer uptake. ^18^F-FDG accumulates not only in tumor cells, but also in macrophages and newly formed granulation tissues, which are infiltrating the marginal areas of tumor necrosis [21].

This study had limitations. Data of the small patient population were retrospectively extracted from medical records of a single university hospital. There were no definite indications for the use of ^18^F-FDG PET imaging in the diagnostic armamentarium of suspected osteomyelitis. Thus, seven of the original 40 patients had PET imaging but never had definite histopathologic or microbiologic verification of the diagnosis probably due to mild symptoms and negative imaging results. The execution of other imaging modalities was not determined but was solely based on the clinical judgment. As a result, imaging studies were not performed in a constant manner for the comparison with ^18^F-FDG PET imaging and the variation could affect the interpretation of ^18^F-FDG PET images. We cannot exclude occasional errors in the sampling of the biopsies as well as in the performance of microbiologic analyses. However, a special attention had been placed to repeat biopsies in culture-negative cases minimizing the risk of errors in surgical sampling. In addition, the microbiologic culture techniques were based on the notion that detection of low-virulent slow-growing bacteria requires extended culture times. Molecular assays, such as polymerase chain reaction (PCR), have been developed to aid in bacterial detection and identification [22, 23]. These techniques were applied, but not in all culture-negative cases. The applied PET imaging technique was constant in all patients and the clinical follow-up time of all patients was long enough, but as a result the imaging was not performed with the current models of PET scanners with a low-dose or full-dose diagnostic CT, which provide means to acquire more precise anatomic and physiologic data improving foremost specificity but also sensitivity [24, 25]. This technical limitation seemed to have only a minor impact, because the interpretation of the PET images of both culture-positive and culture-negative cases was unquestionable and showed high SUV_ratio_ values. Only the two false positive cases due to periarticular soft tissue infections could have been avoided by using ^18^F-FDG PET/CT, because it provides exact anatomic localization of ^18^F-FDG uptake. As a potential technical limitation, a standard circular ROI (15 mm in diameter) was applied in the SUV analyses of FDG tracer accumulation. Certainly, the infected bone regions varied in size and most of them were larger than the selected ROI. The small diameter of the ROI carried a risk for partial-volume effect (PVE) meaning that the apparent pixel values in PET images were influenced by the surrounding high pixel values [26]. If the measured SUV_mean_ values were under the possible influence of PVE, the current analysis included also the comparison of SUV_max_ values based on the maximum uptake of ^18^F-FDG in a single pixel (size of 1.56 × 1.56 mm).

## 5. Conclusion

We conclude that there is no relationship between the level of ^18^F-FDG PET uptake and the presence of positive or negative bacterial cultures in patients with histologically proven osteomyelitis. The result favors the concept that culture-negative cases are false-negative infections due to nonculturable microbes. Thus, ^18^F-FDG PET may help to confirm the presence of active infection in patients with culture-negative low-grade osteomyelitis and guide their appropriate treatment.

## Figures and Tables

**Table 1:** PET-imaged patients with definite histopathologic and/or microbiologic diagnosis (*n* = 26).

Case #	Age/sex	Anatomic location	Implant	Analysis of biopsy sample	Microbiologic culture	Definite diagnosis
1	64/F	Sternoclavicular joint	No	Histology, microbiology	Negative	Osteoarthritis
2	21/M	Femur	No	Histology, microbiology	Negative	Brodie's abscess
3	59/F	Medial clavicle	No	Histology, microbiology	Negative	Chronic osteomyelitis
4	61/F	Sacrum	No	Histology	Not done	Chronic osteomyelitis
5	60/F	Femur	No	Histology, microbiology	Negative	Recurrent chronic osteomyelitis
6	17/F	Humerus	No	Histology, microbiology	Positive	Recurrent chronic osteomyelitis
7	70/M	Pelvis	No	Histology, microbiology	Positive	Chronic osteomyelitis
8	25/M	Tibia	No	Histology, microbiology	Positive	Brodie's abscess
9	67/F	Lumbar region	Yes	Microbiology	Positive	Spinal implant infection
12	21/F	Hip region	No	Histology, microbiology	Positive	Soft tissue infection
13	59/M	Thoracic spine	No	Histology, microbiology	Negative	Vertebral osteomyelitis
15	59/M	Symphysis	No	Histology, microbiology	Positive	Postoperative osteomyelitis
16	52/F	Tibia	No	Microbiology	Positive	Recurrent Brodie's abscess
18	18/M	Thoracic spine	Yes	Microbiology	Positive	Spinal implant infection
20	73/F	Thoracic spine	No	Histology, microbiology	Negative	Vertebral osteomyelitis
25	68/M	Sternum	No	Histology, microbiology	Positive	Soft tissue infection
26	73/F	Thoracic spine	No	Histology, microbiology	Negative	Plasmacytoma
27	19/M	Femur	No	Histology, microbiology	Negative	Recurrent chronic osteomyelitis
29	73/F	Lumbar spine	No	Histology, microbiology	Positive	Vertebral osteomyelitis
30	17/F	Tibia	No	Histology, microbiology	Negative	Brodie's abscess
32	73/F	Elbow region	No	Microbiology	Positive	Soft tissue infection
33	22/F	Ankle region	No	Histology, microbiology	Negative	Soft tissue infection
34	19/F	Tibia	Yes	Histology, microbiology	Positive	Postoperative osteomyelitis
36	42/M	Lumbar spine	No	Histology, microbiology	Negative	Transient bone marrow oedema
39	28/M	Femur	No	Histology, microbiology	Negative	Osteoblastoma
40	42/M	Radius	No	Histology, microbiology	Positive	Chronic osteomyelitis

**Table 2:** Patients with histologically and/or microbiologically proven osteomyelitis (*n* = 16).

Case	Age/sex	Definite diagnosis	Microbiologic culture	Method of biopsy	Antimicrobial therapy before biopsy	Antimicrobial therapy started before PET
6	17/F	Recurrent chronic osteomyelitis of humerus	Coagulase-negative* Staphylococcus*	Open biopsy	No	No
7	70/M	Chronic osteomyelitis of pelvis	*P. aeruginosa, Enterococcus, S. epidermidis*	Open biopsy	Yes, interrupted before biopsy	Yes
8	25/M	Brodie's abscess of tibia	*S. aureus*	Percutaneous biopsy under fluoroscopy	No	No
15	59/M	Postoperative osteomyelitis of symphysis	*S. epidermidis*	MRI-guided biopsy	No	No
16	52/F	Recurrent Brodie's abscess of tibia	*S. aureus*	Open biopsy	No	No
29	73/F	Vertebral osteomyelitis	*S. epidermidis*	Biopsy during laminectomy	No	Yes
34	19/F	Postoperative osteomyelitis of tibia	*S. epidermidis*	Open biopsy and removal of bone screw	No	No
40	42/M	Chronic osteomyelitis of radius	*Bacillus *species	MRI-guided biopsy and repeated open biopsy	Yes, interrupted before biopsy	No

2	21/M	Brodie's abscess of femur	Negative	Repeated open and CT-guided biopsy	No	No
3	59/F	Chronic osteomyelitis of medial clavicle	Negative	Open biopsy	No	No
5	60/F	Recurrent chronic osteomyelitis of femur	Negative	Open biopsy	No	No
13	59/M	Vertebral osteomyelitis	Negative	Biopsy during laminectomy	No	Yes
20	73/F	Vertebral osteomyelitis	Negative	CT-guided biopsy	No	No
27	19/M	Recurrent chronic osteomyelitis of femur	Negative	Repeated MRI-guided and open biopsy	No	Yes
30	17/F	Brodie's abscess of tibia	Negative	MRI-guided and open biopsy	No	No
4	61/F	Chronic osteomyelitis of sacrum	Not done	CT-guided biopsy	No	No

**Table 3:** Results of ^18^F-FDG PET and additional imaging modalities in patients with proven osteomyelitis (*n* = 16).

Case #	Microbiologic culture	PET	MRI	Bone scan	Infection scan	CT	SUV		SUV_ratio_	
							SUV_mean_	SUV_max_	SUV_mean_	SUV_max_
6	Culture-positive	TP	TP	TP	—	—	1.19	1.98	2.25	2.89
7	Culture-positive	TP	FN	TP	FN	—	3.93	5.53	5.44	4.78
8	Culture-positive	TP	TP	TP	TP	TP	0.99	2.86	10.1	20.51
15	Culture-positive	TP	TP	TP	—	TP	4.62	7.59	3.79	5.56
16	Culture-positive	TP	TP	TP	TP	TP	0.90	2.81	3.60	4.36
29	Culture-positive	TP	TP	TP	TP	—	2.54	4.11	2.42	2.83
34	Culture-positive	TP	—	—	TP	FN	1.72	2.59	9.72	11.28
40	Culture-positive	TP	TP	TP	FN	—	1.55	2.37	2.48	2.47

2	Culture-negative	TP	TP	TP	TP	—	0.74	1.16	4.90	2.57
3	Culture-negative	TP	—	TP	—	FN	1.95	2.50	2.30	2.14
5	Culture-negative	TP	TP	TP	TP	—	2.34	2.79	16.10	14.28
13	Culture-negative	TP	TP	—	—	—	3.08	4.43	3.47	3.61
20	Culture-negative	TP	FN	TP	TP	TP	1.80	3.05	2.12	2.77
27	Culture-negative	TP	TP	—	—	—	1.96	2.91	11.44	12.47
30	Culture-negative	TP	TP	TP	TP	—	2.60	2.86	13.20	7.72
4	Not done	TP	FN	TP	TP	FN	2.57	2.80	1.36	1.89

PET = positron emission tomography; MRI = magnetic resonance imaging; Bone scan = three-phase bone scintigraphy; Infection scan = labeled leukocyte scintigraphy; CT = computerized tomography; TP = true positive; FN = false negative; — = not done.

## References

[1] Termaat M. F., Raijmakers P. G. H. M., Scholten H. J., Barker F. C., Patka P., Haarman H. J. T. M. (2005). The accuracy of diagnostic imaging for the assessment of chronic osteomyelitis: a systematic review and meta-analysis. *The Journal of Bone and Joint Surgery—American Volume*.

[2] Prandini N., Lazzeri E., Rossi B., Erba P., Parisella M. G., Signore A. (2006). Nuclear medicine imaging of bone infections. *Nuclear Medicine Communications*.

[3] Wang G.-L., Zhao K., Liu Z.-F., Dong M.-J., Yang S.-Y. (2011). A meta-analysis of fluorodeoxyglucose-positron emission tomography versus scintigraphy in the evaluation of suspected osteomyelitis. *Nuclear Medicine Communications*.

[4] Valle C. D., Parvizi J., Bauer T. W. (2010). Diagnosis of periprosthetic joint infections of the hip and knee. *Journal of the American Academy of Orthopaedic Surgeons*.

[5] Teterycz D., Ferry T., Lew D. (2010). Outcome of orthopedic implant infections due to different staphylococci. *International Journal of Infectious Diseases*.

[6] Lankinen P., Lehtimäki K., Hakanen A. J., Roivainen A., Aro H. T. (2012). A comparative ^18^F-FDG PET/CT imaging of experimental *Staphylococcus aureus* osteomyelitis and *Staphylococcus epidermidis* foreign-body-associated infection in the rabbit tibia. *EJNMMI Research*.

[7] Koort J. K., Mäkinen T. J., Knuuti J., Jalava J., Aro H. T. (2004). Comparative ^18^F-FDG PET of experimental Staphylococcus aureus osteomyelitis and normal bone healing. *Journal of Nuclear Medicine*.

[8] Jones-Jackson L., Walker R., Purnell G. (2005). Early detection of bone infection and differentiation from post-surgical inflammation using 2-deoxy-2-[18F]-fluoro-D-glucose positron emission tomography (FDG-PET) in an animal model. *Journal of Orthopaedic Research*.

[9] White L. M., Schweitzer M. E., Deely D. M., Gannon F. (1995). Study of osteomyelitis: Utility of combined histologic and microbiologic evaluation of percutaneous biopsy samples. *Radiology*.

[10] Floyed R. L., Steele R. W. (2003). Culture-negative osteomyelitis. *Pediatric Infectious Disease Journal*.

[11] Wu J. S., Gorbachova T., Morrison W. B., Haims A. H. (2007). Imaging-guided bone biopsy for osteomyelitis: Are there factors associated with positive or negative cultures?. *American Journal of Roentgenology*.

[12] Heyer C. M., Brus L.-J., Peters S. A., Lemburg S. P. (2012). Efficacy of CT-guided biopsies of the spine in patients with spondylitis - An analysis of 164 procedures. *European Journal of Radiology*.

[13] Sehn J. K., Gilula L. A. (2012). Percutaneous needle biopsy in diagnosis and identification of causative organisms in cases of suspected vertebral osteomyelitis. *European Journal of Radiology*.

[14] Calhoun J. H., Manring M. M., Shirtliff M. (2009). Osteomyelitis of the long bones. *Seminars in Plastic Surgery*.

[15] Gristina A. G., Oga M., Webb L. X., Hobgood C. D. (1985). Adherent bacterial colonization in the pathogenesis of osteomyelitis. *Science*.

[16] Kaim A. H., Weber B., Kurrer M. O., Gottschalk J., Von Schulthess G. K., Buck A. (2002). Autoradiographic quantification of ^18^F-FDG uptake in experimental soft-tissue abscesses in rats. *Radiology*.

[17] Lankinen P. (2013). PET imaging of osteomyelitis—Feasibility of ^18^F-FDG, ^68^Ga-chloride, and ^68^Ga-DOTAVAP-P1 tracers in staphylococcal bone infections. *AnnalesUniversitatisTurkuensis Ser D: Medica Odontologica*.

[18] De Winter F., Van De Wiele C., Vogelaers D., De Smet K., Verdonk R., Dierckx R. A. (2001). Fluorine-18 fluorodeoxyglucose-positron emission tomography: A highly accurate imaging modality for the diagnosis of chronic musculoskeletal infections. *Journal of Bone and Joint Surgery - Series A*.

[19] Murphy R. A., Ronat J.-B., Fakhri R. M. (2011). Multidrug-resistant chronic osteomyelitis complicating war injury in Iraqi civilians. *Journal of Trauma - Injury, Infection and Critical Care*.

[20] Johnson E. N., Burns T. C., Hayda R. A., Hospenthal D. R., Murray C. K. (2007). Infectious complications of open type III tibial fractures among combat casualties. *Clinical Infectious Diseases*.

[21] Kubota R., Yamada S., Kubota K., Ishiwata K., Tamahashi N., Ido T. (1992). Intratumoral distribution of fluorine-18-fluorodeoxyglucose in vivo: High accumulation in macrophages and granulation tissues studied by microautoradiography. *Journal of Nuclear Medicine*.

[22] Fenollar F., Roux V., Stein A., Drancourt M., Raoult D. (2006). Analysis of 525 samples to determine the usefulness of PCR amplification and sequencing of the 16S rRNA gene for diagnosis of bone and joint infections. *Journal of Clinical Microbiology*.

[23] Fihman V., Hannouche D., Bousson V. (2007). Improved diagnosis specificity in bone and joint infections using molecular techniques. *Journal of Infection*.

[24] Von Schulthess G. K., Steinert H. C., Hany T. F. (2006). Integrated PET/CT: Current applications and future directions. *Radiology*.

[25] Strobel K., Stumpe K. D. M. (2007). PET/CT in musculoskeletal infection. *Seminars in Musculoskeletal Radiology*.

[26] Hoffman E. J., Huang S.-C., Phelps M. E. (1979). Quantitation in positron emission computed tomography: 1. effect of object size. *Journal of Computer Assisted Tomography*.

